# Modern CRRT systems are associated with lower risk of hypothermia

**DOI:** 10.1038/s41598-024-74977-2

**Published:** 2024-10-05

**Authors:** Max Bell, Daniel Hertzberg, Fredrik Hansson, Åsa Carlsson, Johan Berkius, Laszlo Vimlati, Ann-Kristin Nilsson, Carolina Ekström, Marcus Broman

**Affiliations:** 1 Department of Physiology and Pharmacology, Karolinska Institutet, Stockholm, Sweden; 2 Perioperative Medicine and Intensive Care, Karolinska University Hospital, Stockholm, Sweden; 3 IRLAB, Integrated Research Laboratories Sweden AB, Gothenburg, Sweden; 4 Sunderby Sjukhus, Luleå, Sweden; 5 Department of Anaesthesiology and Intensive Care, Västervik Hospital, Västervik, Sweden; 6 Department of Cardiothoracic Surgery, Uppsala University Hospital, Uppsala, Sweden; 7 Perioperative and Intensive Care, Skåne University Hospital, Lund, Sweden

**Keywords:** AKI, Blood warming devices, Critical illness, CRRT, Hypothermia, Biophysics, Physiology

## Abstract

One risk of continuous renal replacement therapy (CRRT) is inadvertent hypothermia (IH), which is defined as a non-therapeutic core temperature decrease below normal. In continuous renal replacement therapy, heat loss will always occur from blood pumped through the dialysis circuit to cooler environment, predisposing for hypothermia. Blood flow and effluent flows are the most important parameters causing heat loss. We investigated and compared the novel TherMax warmer to previous generation technologies during CRRT in a multicenter setting. This was a prospective observational multicenter study with historic single-center controls. The study group consisted of 100 patients in eight Swedish ICUs with clinical indication for CRRT, using the PrisMax platform and TherMax warmer. Both patient and set warmer temperatures were recorded hourly for the first 24 h. The presence of treatment hours in hypothermia (< 36.0 Celsius) and the difference between set warmer temperature and measured patient temperature in the multi-center study cohort were compared to a matched single-center historic control cohort treated with the old Prismaflex platform and adjacent Barkey warmer. In the TherMax group 77/100 (77.0%) of patients, and for controls 26/86 (30.2%) of patients were free of hypothermia (Chi square, *p* < 0.001). The mean number of hours spent in hypothermia was (mean ± SD) 0.66 ± 1.60 and 6.92 ± 7.79 h in the TherMax and control groups, respectively (Chi square *p* < 0.001). In the study group the patient temperature was higher than the set temperature on the warmer with a difference of Δ0.47 ± 0.80 °C (minor difference), whereas in the control group the set temperature on the warmer was higher than the patient temperature with a difference of Δ4.55 ± 1.00 °C (over-correction). The novel TherMax warmer technology protected against hypothermia and was significantly more accurate than the Barkey warmer.

## Introduction

A common risk of continuous renal replacement therapy (CRRT) is inadvertent hypothermia (IH). A single-center study from 2021 found hypothermia in over 50% of CRRT cases^1^. Another study reported severe hypothermia (< 35.0 °C), in over 4/10 patients^2^, where a previous study from this group found that 11.43% and 10.06% of time during CRRT was spent below 36 °C in patients using the Prismaflex and PrisMax CRRT systems, respectively^3^. Data on this subject are still scarce and are dominated by case reports^4^. A recent review of complications associated with CRRT listed hypothermia as one where preventive strategies exist^5^.

Inadvertent hypothermia is defined as an uncontrolled, nontherapeutic core temperature decrease below normal, inadvertently induced in a therapeutic setting. Unlike fever, which regulates core temperature elevation in response to noninfectious or infectious stimuli, IH generally develops secondary to heat loss, cold environment exposure, and/or dysfunction of normal thermoregulatory mechanisms^6^. In contrast to fever, IH lacks any adaptive value, and even a mild core temperature decrease below normal can result in severe complications^7^. Inadvertent hypothermia has been extensively investigated in perioperative settings. A recent study on lung surgery found that patients experiencing inadvertent perioperative hypothermia had higher risk-adjusted rates of overall morbidity and infectious postoperative complications^8^. Another investigation reported an intraoperative incidence of intraoperative hypothermia of 73.5% and postoperative hypothermia of 11.9%^9^. Focusing strictly on *postoperative*IH events, data show that patients with temperatures < 36 °C are at greater risk for surgical site infection, increased mortality, longer length of hospital stay, and higher 30-day readmission rates^10^. In critically ill patients, a systematic review on inadvertent hypothermia showed that lowest core temperature was independently associated with significantly higher mortality. High severity and a long duration of hypothermia are also associated with higher mortality. Mortality was significantly higher in patients with a core temperature < 36.0 °C (odds ratio (OR) 2.1) and at temperatures < 35.0 °C (OR 2.9)^11^.

The rate at which blood is pumped through the dialysis circuit affects heat loss, but the relationship is complex. With higher blood flow, more blood meets the colder environment, but warmer blood is inserted into the circuit, and the blood-environment exposure is shorter. However, the effluent flow corresponds proportionally to the increased heat loss because blood meets the increased volume of (non-heated) treatment fluids in the filter.

Very few studies have investigated whether modern blood warmers used during CRRT can avoid or minimize inadvertent hypothermia. Our previous study compared the novel TherMax blood warmer in a single-center setting^3^. This prospective cohort study across multiple ICUs across Sweden assessed whether single-center findings would hold up: how common is inadvertent hypothermia during CRRT? Is the newer blood warmer associated with a lower risk of inadvertent hypothermia than the previous generation of blood warmers for the Prismaflex system?

## Materials and methods

### Ethics

This prospective observational multicenter study used historic single-center controls. The Swedish Ethical Review Authority approved the study (dnr 2022-03204-01) and waived the need for informed consent, all research was performed in accordance with relevant guidelines/regulations. ClinicalTrials.gov Identifier: NCT03973814, 04/06/2019.

### Patients and data collection

Inclusion criteria were adult patients aged 18 years or older with manifest or developing AKI and indication for CRRT. This *prospective study group* consisted of 100 unique patients within the participating Swedish ICUs. The inclusion period ran from September 2020 to September 2023.

We studied the first 24 h of CRRT using the PrisMax platform and the TherMax warmer. The *control group*, using the Prismaflex platform with the Barkey warmer, consisted of 86 matched patients selected from 310 screened patients treated in the adult general ICU at Skåne University Hospital, Lund, Sweden during 2014–2023. The matching of controls was based on having at least 25 h-by-hour temperature measurements from one hour before CRRT initiation and onwards.

In the prospective study group, the hourly set temperature on the TherMax warmer was noted, and the simultaneously measured patient temperature was recorded. In the matched control group, corresponding data were extracted from the electronic medical records system (ICCA, Philips, Vienna, Austria). Comorbid conditions, reason for ICU admission and indication for CRRT were collected and managed using REDCap electronic data capture tools (https://www.project-redcap.org/) hosted at Karolinska Institutet.

A total of eight ICUs in Sweden participated in the study: Skåne University Hospital, Lund, 26 patients; Karolinska University Hospital Central ICU, Stockholm, 25 patients; Sunderbyn Hospital, Luleå, 15 patients; Karolinska University Hospital Cardiothoracic ICU, Stockholm, 11 patients; Uppsala University Hospital Cardiothoracic ICU, Uppsala, 9 patients; Västervik Hospital, Västervik, 7 patients; Norrköping Hospital, Norrköping, 5 patients; Karolinska University Hospital, Huddinge unit, 2 patients.

### Statistics

The SAS Statistical Analysis Software (Statistical Analysis Software, North Carolina, USA) was used for statistical analysis. All data are presented using descriptive statistics. The number of hours below 36 degrees Celcius was analyzed using the Chi-Square test without continuity correction and Student t-test. The hourly mean difference starting from one hour before CRRT initiation to 24 h after initiaton is presented with a graph. The difference in hours spent in hypothermia was analyzed using the Wilcoxon test and Student t-test. The mean differences are presented with 95% confidence intervals. Statistical significance was set at *p* < 0.001.

## Results

Table 1 details the demographics of the TherMax study group, and the matched historical subset of patients, treated with the older CRRT- and warming systems. Compared to the controls, the prospective PrisMax study group started CRRT at higher renal biomarker levels. Patient temperature differed slightly, but set CRRT-warmer temperature differed significantly. Statistical significance was set to < 0.001.

**Table 1:** Baseline clinical data for the study group and the historic control group. The historic control group (*n* = 86) was matched from a cohort consisting of 310 CRRT patients at Skåne University Hospital, Lund, Sweden and was not originally designed for this study and therefore some data is missing.

Patient data	**Historic control group** *n* = 86	**Study group** *n* = 100	*p* value (student t test)
Gender	**female** 37 (43.0%)	**female** 64 (64.0%)	0.492
Age	**mean** 64.0 years **SD** 13.5 years	**mean** 59.9 years **SD** 14.1years	0.052
Urea at dialysis start	**mean** 17.47 mmol/L **SD** 12.08 mmol/L	**mean** 22.65 mmol/L **SD** 13.65 mmol/L	0.009
Creatine at dialysis start	**mean** 252.8 µmol/L **SD** 202.7 µmol/L	**mean** 311.9 µmol/L **SD** 185.5 µmol/L	0.047
KDIGO class at dialysis start	**no AKI** 17.4% **KDIGO 1** 5.6% **KDIGO 2** 14.2% **KDIGO 3** 62.8%	**no AKI** 7.0% **KDIGO 1** 17.2% **KDIGO 2** 13.1% **KDIGO 3** 62.7%	0.052
Temperature data	**Historic control group** *n* = 86	**Study group** *n* = 100	*p* value (student t test)
Patient temperature at dialysis start	**mean** 36.49 °C **SD** 1.04 °C **median** 36.55 °C **Q1** 36.00 °C **Q3** 37.00 °C	**mean** 37.11 °C **SD** 1.19 °C **median** 37.00 °C **Q1** 36.60 °C **Q3** 37.80 °C	< 0.001
CRRT machine warmer set temperature at dialysis start	**mean** 40.9 °C **SD** 0.83 °C **median** 41.00 °C **Q1** 41.00 °C **Q3** 41.00 °C	**mean** 36.93 °C **SD** 0.41 °C **median** 37.00 °C **Q1** 37.00 °C **Q3** 37.00 °C	< 0.001
CRRT indication; several indications apply for a single patient	**Historic control group** *n* = 86	**Study group** *n* = 100	*p* value (student t test)
Anuria	no data available	46 (46%)	
Sepsis	no data available	40 (40%)	
Fluid removal, extra-vascular	no data available	38 (38%)	
Metabolic acidosis	no data available	28 (28%)	
Uremia	no data available	26 (26%)	
Oliguria	no data available	26 (26%)	
Cardiac	no data available	21 (21%)	
Hyperkalemia	no data available	22 (22%)	
Postoperative	no data available	17 (17%)	
Fluid removal, pulmonary edema	no data available	10 (10%)	
Hepato-renal	no data available	7 (7%)	
Hypovolemia-prerenal	no data available	5 (5%)	
Dysnatremia	no data available	5 (5%)	
Endotoxin removal	no data available	4 (4%)	
Rhabdomyolysis	no data available	5 (5%)	

**Figure 1:**
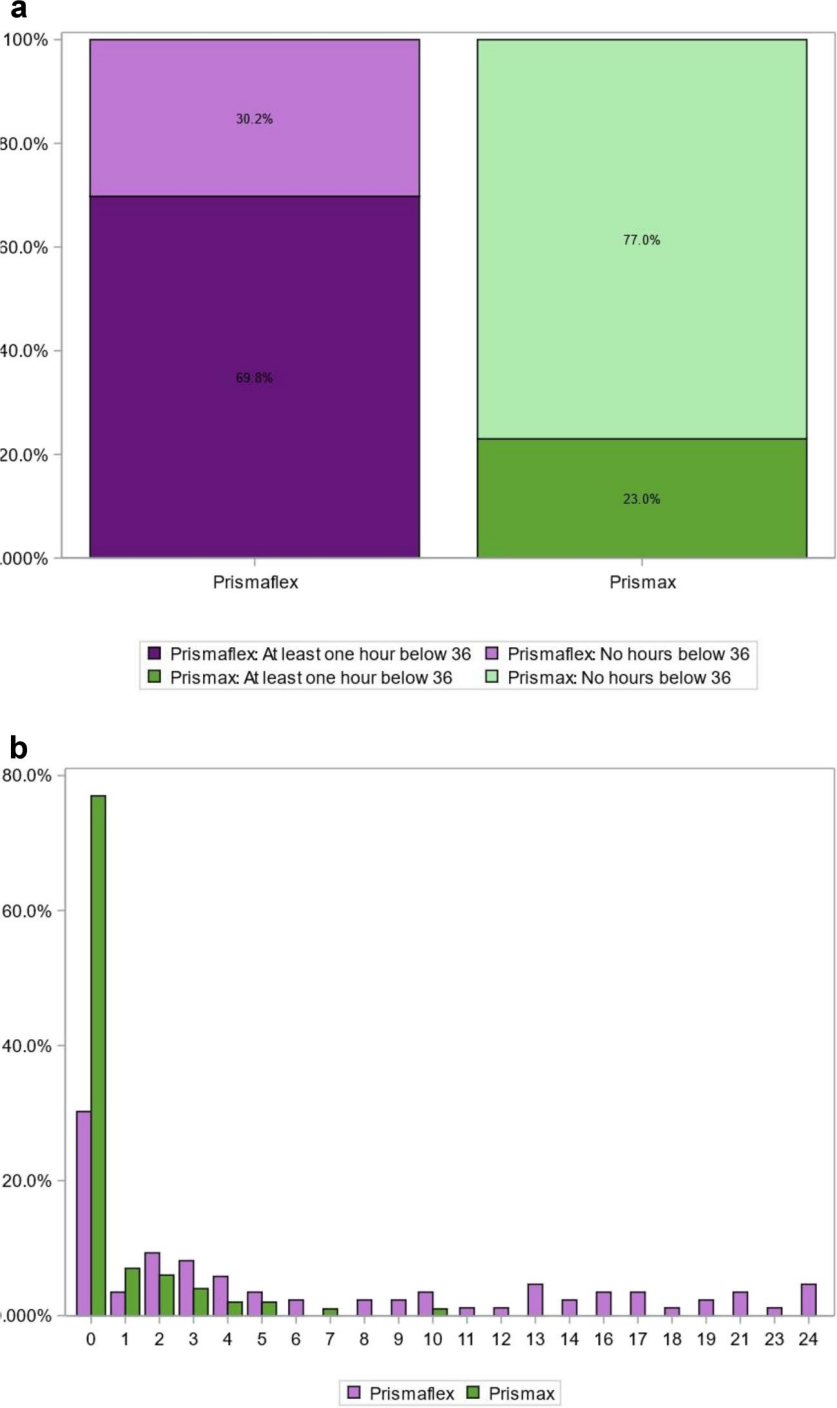
**a** The percentage of patients with zero treatment hours in hypothermia (in lighter hue of green and purple) and at least one treatment hour in hypothermia (in darker hue of green and purple) for the old Barkey control group (Prismaflex platform) and for the TherMax study group (PrisMax platform). Hypothermia is defined as a patient temperature reading of < 36.0 °C for the actual treatment hour. **b** The percentages of the patients in the study- (green) and control- (purple) groups with 0–24 h in hypothermia (< 36.0 °C). A majority (77.0%) of the study group had 0 hypothermia hours. There was a larger fraction of patients with 1–24 h in hypothermia in the control group compared to the study group (Chi square, *p*  < 0.001).

Figure 1a shows the crude shares of patients *without treatment hours in hypothermia during the 24 h study period*; for the PrisMax study group 77/100 (77.0%, 95% CI; 68.8–85.2%) and the matched historic controls: 26/86 (30.2%, 95% CI; 20.5–39.9%) patients (Chi square, *p* < 0.001).

Figure 1b shows the percentages of the patients in the study- and control groups with 0–24 h in hypothermia. A majority of the patients (77.0%) in the study group did not have hypothermia hours at all. There was a lower fraction of patients with 1–24 h in hypothermia in the study group compared to the control group (Chi square, *p* < 0.001).

*The total CRRT-treatment hours spent in hypothermia* during the 24 h study period were mean ± SD 0.66 ± 1.60 h in the study group and mean ± SD 6.92 ± 7.79 h, in the control group (Wilcoxon, *p* < 0.001, Student t test, *p* < 0.001).

**Figure 2:**
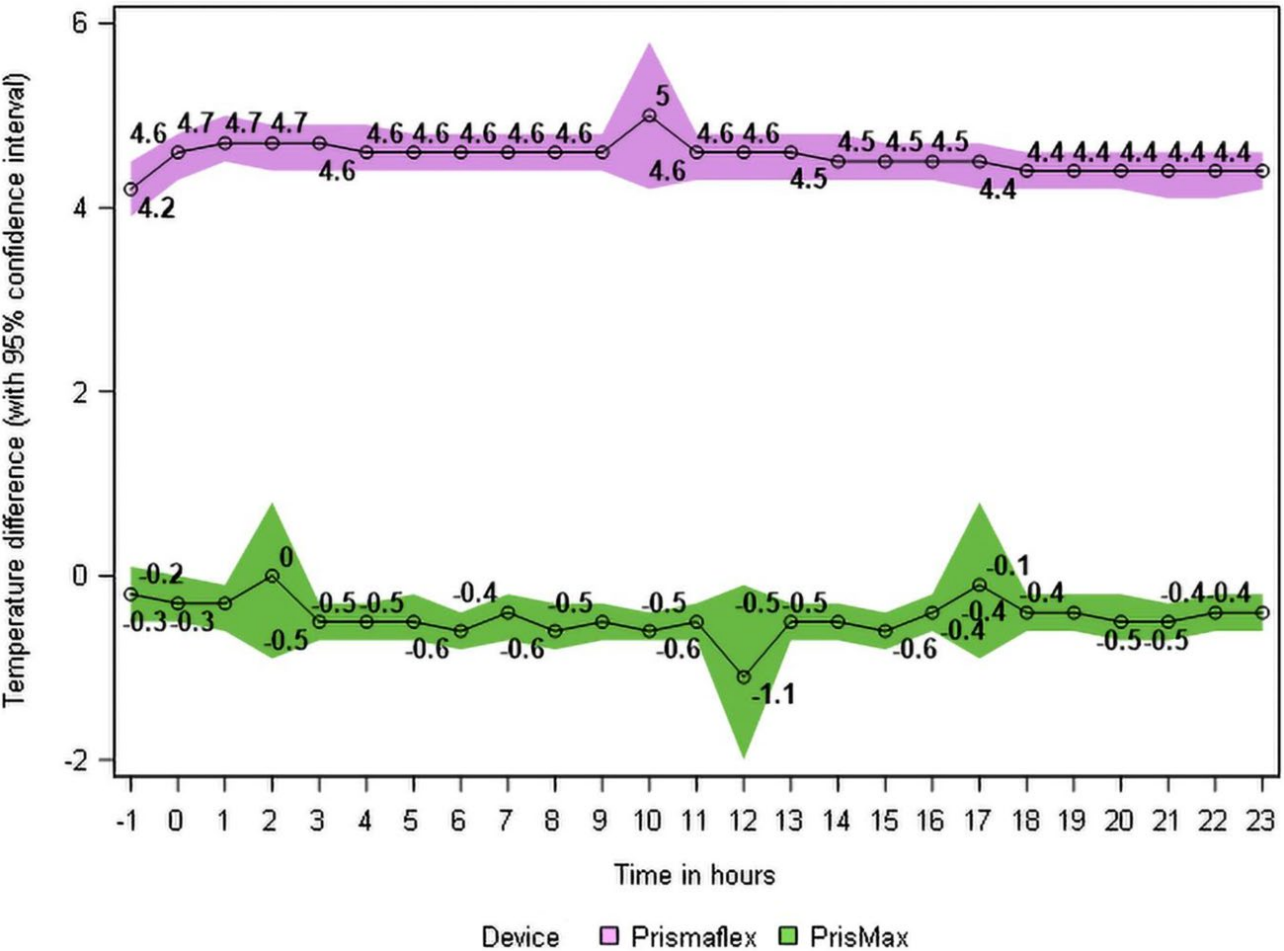
The truncated graphs of the difference between the set temperature on the warmer and the real simultaneous temperature in the patient (if the set temperature on warmer and the temperature in patient eqals the difference will be zero) are shown for the complete 24 h study period in the study group (green, PrisMax platform with warmer TherMax) and the control group (purple, Prismaflex platform with warmer Barkey). The TherMax warmer is more efficient in achieving the set temperature in the patient, and therefore the difference is close to zero, compared to the old Barkey warmer, where overadjustment was used.

The mean ± SD difference between [set temperature on warmer - measured temperature in the patient] was − 0.47 ± 0.80 °C in the study group (warmer set temperature slightly lower than the patient temperature), versus + 4.55 ± 1.00 °C (warmer set temperature higher than the patient temperature) in the control group, during the complete 24 h study period (Wilcoxon, *p* < 0.001, Student t test, *p* < 0.001). A difference of zero would mean that the set temperature on warmer and the measured temperature in the patient match completely. The truncated graphs displaying these differences between [set temperature on warmer - measured patient temperature] are shown in Fig. 2.

## Discussion

This prospective multicenter study in Swedish ICUs examined the temperature of critically ill patients treated with continuous renal replacement therapy. We found that inadvertent hypothermia is uncommon among users of the latest generation of blood warmers. On average, patients treated with modern equipment spent less than one hour below 36.0 °C as compared to almost seven hours under 36.0 °C in patients using the previous generation warmer. The latest generation of blood warmers was also more exact than the older systems, where “over-correction” was needed to avoid hypothermia.

In most cases, body temperature is not the cause, but rather the indicator of the severity, and thus the outcome of the disease. Hypothermia can be used for therapeutic benefit, such as induced hypothermia after cardiac arrest. In a systematic review and meta-analysis of high-quality randomized trials of patients with traumatic brain injury, serious infections, and stroke, the authors reported that therapeutic hypothermia is associated with higher mortality and no difference in good neurologic outcomes compared with normothermia in these groups of critically ill patients^12^. Inadvertent hypothermia, both in the perioperative setting and among critically ill patients, has consequences: shivering and increased catecholamine secretion cause vasoconstriction and elevated heart, respiratory, and metabolic rates; that can lead to caloric losses. These physiologic changes increase risk of myocardial ischemia in patients with coronary heart disease, secondary to decreased coronary blood flow and increased need of myocardial oxygenation^13,14^. Moreover, IH inhibits normal coagulation by decreasing fibrinolytic activity and inhibiting the normal function of platelets and clotting factor enzymes, leading to increased blood loss and transfusion requirements^15,16^. IH is associated with impaired wound healing and an increased risk of surgical infections, partly caused by local tissue vasoconstriction and suppression of immune system activity^17,18^. Laupland and co-workers found severe hypothermia to increase risk for subsequent ICU-aquired infection^19^, but results are confilicting^20^. Some animal studies indicate that naturally occurring hypothermia may be advantageous^21^and in human sepsis patients spontaneous hypothermia was transient, self-limiting and nonterminal^22^. However, induced hypothermia in patients with sepsis was tested in a randomized study that was stopped early for futility^23^. Spontaneous hypothermia in sepsis occurs in 15–35% of patients and is associated with higher mortality than during normothermia or fever^24–29^. In a meta-analysis of 42 clinical trials and over 10,000 patients, reporting body temperature and mortality in patients with sepsis, fever was associated with reduced mortality and hypothermia was associated with increased mortality^24^. However, as the authors state, this association does not imply that fever is always beneficial and hypothermia harmful among septic patients^30^. In patients with hypothermia during sepsis, active warming is common according to a survey study published in 2020^31^. A small randomized pilot study of afebrile patients with sepsis indicated that therapeutic hyperthermia was beneficial in terms of improved mortality in the intervention group^32^.

In line with these findings, there have been studies on how to avoid IH in the critically ill. A UK study showed some improvement following educational efforts and the introduction of a clinical protocol^33^.

In a previously mentioned single-center cohort study of 186 patients, the incidence of hypothermia, defined as a body temperature ≤ 35 °, was 52.7%^1^. The incidence was significantly higher in septic shock patients (relative risk 2.11) and in those who underwent hemodiafiltration (relative risk 1.50) with heating in the return line, using the Prismaflex device and Prismaflo heating system^1^. The present study prospectively collected data from a range of Swedish ICUs using the novel PrisMax CRRT system and TherMax warmer. In contrast to all other publications, except our previous single-center study^3^, we report data on multiple measurements of inadvertent hypothermia.

Limitations exist. We lack data on use of parallel warming methods and inaccuracies in temperature readings can exist, however, these issues were likely just as prevalent among the historic controls. Baseline body temperature was slightly lower among historic controls. We do not have room temperature data. We lack data on the blood flow and effluent flow. However, the old Barkey system (control group) prevents cooling by reversing the gradient between the blood compartment and the ambient air by simply maintaining a constant preset temperature in a warming sock covering the tubing. Thermax (study group) has a 27 ml bag through which the blood passes and where warming takes place based on preset temperature (operator’s input), actual blood temperature and blood flow. The intensity of the warming process in the bag will be adjusted via a closed loop. Therefore, the Thermax warmer is blood flow independent. The Thermax warmer can cope with the blood flow range available on the Prismax machine 0-450 ml/min. Strengths include the fact that data were prospectively collected from multiple intensive care units across the nation. These ICUs treat a wide range of patients; indicating that these results – lower risk of inadvertent hypothermia – are generalizable in industrialized countries. Our findings indicate that clinicians may avoid therapy induced IH by using the latest CRRT technology, but further large scale studies are needed to evaluate if this translates to improved outcomes.

## Conclusion

In critically ill patients treated with CRRT, the TherMax warmer technology minimized the time spent below 36.0 °C and thus protects against inadvertent hypothermia. The novel warmer was significantly more precise than the old Barkey warmer.

## Data Availability

The datasets used during the current study are available from the corresponding author on reasonable request.

## Declarations

The authors Bell and Broman have an IIR Grant from Baxter Medical.
